# Translational Relevance of Secondary Intracellular Signaling Cascades Following Traumatic Spinal Cord Injury

**DOI:** 10.3390/ijms25115708

**Published:** 2024-05-24

**Authors:** Mohammad-Masoud Zavvarian, Akshat D. Modi, Sarah Sadat, James Hong, Michael G. Fehlings

**Affiliations:** 1 Division of Genetics and Development, Toronto Western Hospital, University Health Network, Toronto, ON M5T 2S8, Canada; mohammad.zavvarian@mail.utoronto.ca (M.-M.Z.); akshat.modi@mail.utoronto.ca (A.D.M.); sarah.sadat@mail.utoronto.ca (S.S.); james.hong@uhn.ca (J.H.); 2 Institute of Medical Science, Faculty of Medicine, University of Toronto, Toronto, ON M5S 1A8, Canada; 3 Department of Biological Sciences, University of Toronto, Scarborough, ON M1C 1A4, Canada; 4 Department of Human Biology, University of Toronto, Toronto, ON M5S 3J6, Canada; 5 Department of Surgery, Faculty of Medicine, University of Toronto, Toronto, ON M5T 1P5, Canada

**Keywords:** spinal cord injury, intracellular signaling, protein kinases, kinome, MAPK, PI3K-AKT-mTOR, Rho-ROCK, NF-κB, JAK-STAT

## Abstract

Traumatic spinal cord injury (SCI) is a life-threatening and life-altering condition that results in debilitating sensorimotor and autonomic impairments. Despite significant advances in the clinical management of traumatic SCI, many patients continue to suffer due to a lack of effective therapies. The initial mechanical injury to the spinal cord results in a series of secondary molecular processes and intracellular signaling cascades in immune, vascular, glial, and neuronal cell populations, which further damage the injured spinal cord. These intracellular cascades present promising translationally relevant targets for therapeutic intervention due to their high ubiquity and conservation across eukaryotic evolution. To date, many therapeutics have shown either direct or indirect involvement of these pathways in improving recovery after SCI. However, the complex, multifaceted, and heterogeneous nature of traumatic SCI requires better elucidation of the underlying secondary intracellular signaling cascades to minimize off-target effects and maximize effectiveness. Recent advances in transcriptional and molecular neuroscience provide a closer characterization of these pathways in the injured spinal cord. This narrative review article aims to survey the MAPK, PI3K-AKT-mTOR, Rho-ROCK, NF-κB, and JAK-STAT signaling cascades, in addition to providing a comprehensive overview of the involvement and therapeutic potential of these secondary intracellular pathways following traumatic SCI.

## 1. Introduction

A traumatic spinal cord injury (SCI) has a devastating impact on a patient’s independence, lifestyle, and socioeconomic status [1]. The prevalence of SCI ranges from 250 to 906 cases per million and constitutes the second-leading cause of paralysis worldwide [2]. The life expectancy of traumatic SCI patients often spans several decades from the time of injury, leading to a poor quality of life and lifelong disability. Additionally, the estimated lifetime cost of living with traumatic SCI paralysis can exceed USD 5,000,000 per individual, which introduces significant financial difficulties to the patient’s family and healthcare providers [3]. However, despite the high prevalence, severity, and repercussions of traumatic SCI, treatment options in the clinic continue to be limited [4].

In a healthy individual, the spinal cord transmits and processes neuronal signals between the brain and peripheral organs through a complex network of ascending and descending tracts as well as spinal interneurons [5]. These cells are supported by a variety of glial and vascular cells, including astrocytes, oligodendrocytes, microglia, fibroblasts, pericytes, endothelial cells, and smooth muscle cells [6,7,8]. However, following trauma to the spinal cord, either by compression, contusion, or laceration, there is mechanical damage to neuronal cell bodies, axonal tracts, blood vessels, and their surrounding glial cells. Referred to as the primary injury, this mechanical damage also initiates a subsequent cascade of pathobiological events known as the secondary injury, characterized by the production of toxic cellular debris [9,10], disruption of the local microvasculature [11], compromised integrity of the blood–spinal cord barrier (BSCB) [12], and initiation of a neuroinflammatory response [13,14,15,16,17] that further exacerbates damage to the neuronal pathways [18]. Although the clinical manifestation of these pathologies is heterogeneous, they often result in sensory loss and flaccid paralysis due to the loss of peripheral innervation at and distal to the injury site, in addition to autonomic disturbances, such as neurogenic shock and autonomic dysreflexia [19,20,21]. Damage to spinal interneurons, such as in central cord syndrome, can also result in pain, spasticity, or functional inactivation [22].

The cellular responses behind these secondary pathologies are mediated through a series of intracellular signaling cascades that can present promising therapeutic targets to attenuate further damage or even induce regenerative effects to enhance functional recovery [23]. Due to the complex and integrated nature of intracellular signaling, the role of these pathways and their underlying protein kinases following traumatic SCI are elusive targets for clinical translation. However, recent advances in kinomics, transcriptional, and proteomics techniques, as well as a plethora of pharmacological investigations, have enhanced our understanding of these intracellular processes and their therapeutic potential for traumatic SCI. This narrative review aims to survey the recent literature on the signal transducers involved in secondary SCI pathogenesis and their pharmacological targets to enhance recovery following traumatic SCI.

## 2. Secondary SCI Pathobiology

**Figure 1:**
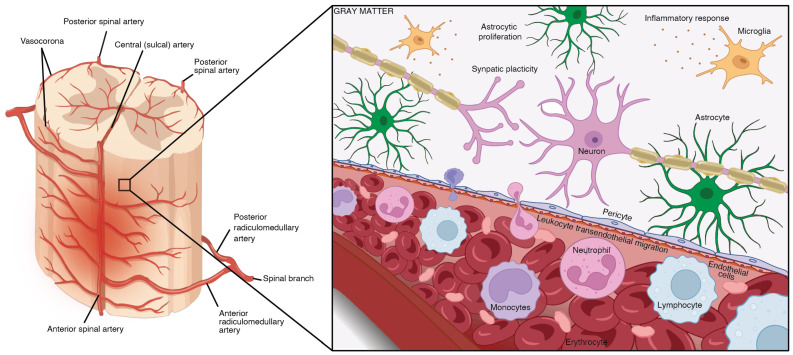
The early pathogenesis of secondary traumatic SCI at the lesion core. The BSCB is crucial for maintaining the stability of neural tissue by selectively preventing the transfer of ions between the circulatory and nervous systems. Traumatic SCI compromises the integrity of this barrier, leading to increased permeability, swelling, ischemia, pro-apoptotic signaling, and the introduction of pro-inflammatory immune cells and cytokines. The spinal vasculature on the left was adapted and edited with permission from Santillan et al. [44], 2012, BMJ Publishing Group LTD, and the schematic was created in Biorender (BioRender.com) with permission.

The compressive-contusive damage to the spinal cord permeabilizes the cellular membranes and disrupts the integrity of the spinal microvasculature, leading to ischemia, hemorrhage, and the release of cellular debris [24,25]. The resident microglia and astrocytes then trigger a greater immune reaction by recruiting blood-borne immune cells to clear the oxidative species and free radicals [26] (Figure 1). While the neuroinflammatory response serves an important function in regulating tissue damage following the initial trauma, prolonged inflammation leads to a secondary SCI pathogenesis that exacerbates functional loss. The secondary response to traumatic SCI involves a variety of neuronal populations as well as glial cells, such as astrocytes, microglia [27], fibroblasts [28], oligodendrocytes [29,30], pericytes [31,32], and circulatory immune cells [33].

The progression of secondary pathogenesis after traumatic SCI is categorized into four phases: acute (within 48 h), subacute (2 to 14 days), intermediate (14 to 56 days), and chronic (beyond 56 days) [18]. The impaired microvascular network restricts the blood flow at the lesion core in the acute phase of injury, leading to the induction of hypoxia, ischemic injury, and hemorrhage [12,34]. Following pro-inflammatory signals from the resident microglia and astrocytes, reactive circulatory cells and molecules infiltrate the neural tissue [35]. In the subacute phase, BSCB integrity is re-established, and scar-forming astrocytes begin to proliferate near the lesion to encapsulate the infiltrated immune cells [36,37,38]. Astrocytes have multiple functions in the injured spinal cord and play an important role in organizing and maintaining the injured spinal cord [36,38,39]. In parallel, myelin sheath damage and demyelination result in inhibitory molecules that get deposited around the astrocytic border. Within the intermediate phase, local cellular reactions continue. Ultimately, during the chronic phase, the extended remodeling and secondary cellular response lead to the development of cystic cavities, Wallerian degeneration, and neuroplasticity [25].

These secondary SCI pathologies result in the formation of three distinct histological sections in the injured spinal cord [26]. These histological compartments consist of an injury epicentre characterized by cavitation and fibrotic scar [31,40,41], an astrocytic border surrounding the injury epicentre [38,42], and an adjacent perilesional zone of neural tissue, in which circuit remodeling and neuroplasticity occur [13,26]. Each of these three histologically distinct compartments is formed throughout the course of secondary SCI pathogenesis and presents unique challenges for regeneration and recovery [43].

At the injury epicenter, debris clearance leads to the formation of cavitations that limit axonal growth and sprouting [45,46]. In addition, fibroblasts proliferate and migrate from nearby perivascular sites to the lesion site, as well as enclosing a large number of macrophages, leading to fibrotic scar formation [40,47,48]. Fibroblasts are spindle-shaped, progenitor mesenchymal cells involved in extracellular matrix homeostasis, tissue metabolism, the generation of mechanical force and signaling factor secretion, which aid in tissue synthesis, the creation of stem cell signaling niches, and the regulation of innate as well as adaptive immunity [49]. The SCI-induced fibrotic scar forms a biomechanical barrier by releasing EphB2, neural/glial antigen 2 (NG2) proteoglycans, Semaphorin 3A and tenascin C, which inhibit axon regeneration and functional recovery [47,50,51].

Immediately adjacent to the non-neural lesion core, the newly proliferated astrocytes migrate and intermingle with chondroitin sulfate proteoglycans (CSPGs) to generate a barrier, referred to as the astrocytic border [36,52]. Serum proteins and local cell markers, including ATP, sonic hedgehog (SHH), bone morphogenic proteins (BMPs), thrombin, fibroblast growth factor 2 (FGF2), and endothelin, are known mediators that promote astrocyte proliferation [53,54,55,56,57]. These proliferating astrocytes then synthesize and secrete CSPGs, via NG2+ oligodendrocyte progenitor cells (NG2-OPC) into the extracellular matrix [36].

The perilesional zone of spared neural tissue extends away rostro-caudally from the astrocytic border and contains reactive astrocytes, microglia, and NG2-OPCs that induce spontaneous synaptic plasticity and circuit reorganization [27]. In contrast to the newly formed astrocytes in the astrocytic border, the hypertrophic reactive astrocytes in the perilesional zone maintain their connections with local neurons and are directly involved in synaptic regulation. The activity of reactive glial cells as well as circuit reorganization gradually reduces and transitions into healthy spinal cord tissue [26].

Each of these SCI-induced histological compartments presents unique challenges and opportunities for therapeutic intervention. Several intracellular cascades have been shown to be involved in the progression of secondary SCI pathogenesis. These include the MAPK network, the PI3K-AKT-mTOR network, the Rho-ROCK pathway, the NF-κB pathway, and the JAK-STAT pathway. These signaling cascades control a wide range of cellular processes involved in neuroinflammation, scar formation, glial response, and neuroplasticity. Targeting these secondary cellular mediators presents a translationally relevant therapeutic strategy, as they are highly ubiquitous in different cell populations and are well preserved across eukaryotic species. This review article will outline these intracellular pathways and survey the pharmacological interventions that have targeted these cascades with great success.

## 3. MAPK Signaling Network

The MAPK signaling network is comprised of complex interacting cascades with constant crosstalk that are regulated by factors involved in cell proliferation, growth, survival, stress response, immune defense, and apoptosis [58,59]. MAPK signaling has been demonstrated to be involved in many SCI-induced cellular processes, including immune response, scar formation, and neuronal regulation [60,61,62,63,64]. The current clinically approved neuroprotective regimen, involving the administration of methylprednisolone sodium succinate (MPSS), is known to suppress the immune response through alteration of the MAPK network [65,66,67,68]. Interestingly, a recent comparative cross-species transcriptional analysis has highlighted the therapeutic potential of MAPK signaling in traumatic SCI [22]. The scarless healing process post-SCI seen in regenerative species, such as salamanders, may be explained by the downregulation of MAPK orthologs. Due to the observation that these genes are upregulated in mammals and downregulated in salamanders post-SCI, they may be worth investigating as potential therapeutic targets [22].

**Figure 2:**
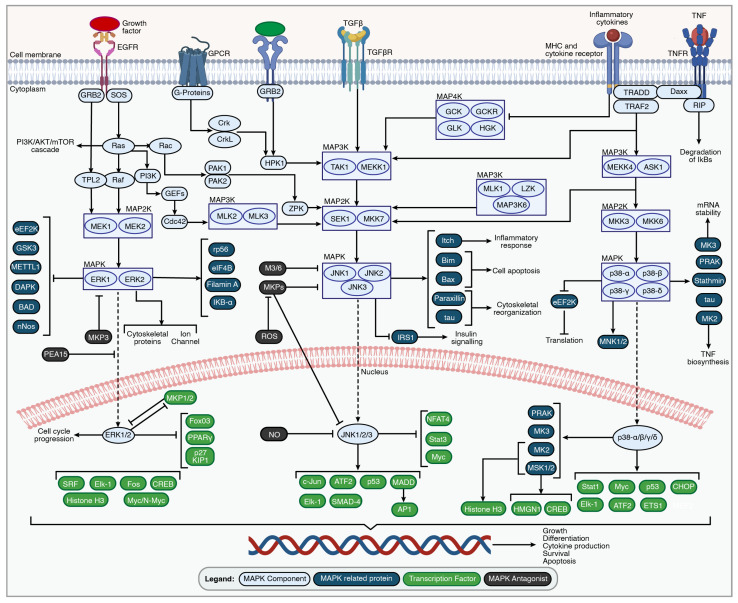
An overview of the MAPK signaling pathways. MAPK signaling cascades are activated by the stimulation of the external growth factor receptor (EGFR), G-protein-coupled receptors (GPCRs), transforming growth factor beta (TGFβ) receptor, tumor necrosis factor receptors (TNFRs), MHC, and cytokine receptors through growth factors, inflammatory cytokines, and environmental stressors. The set of adaptors (GRB2, Crk, and TRAF2) links the activated receptor to guanine nucleotide exchange factors (SOS and HPK1), leading to signal transduction through small GTP-binding proteins such as Ras, Rac, PAK1/2, and ZPK. The signal is transmitted through specific receptor-associated MAP3K, MAP2K, and MAPK family members. The unique MAP3K-promoting MAP4K family members are inhibited during the activation of MAPK pathways due to inflammation or environmental stress. The activated MAPK family members such as ERK1/2, JNK1/2/3, and p38α/β/γ/δ translocate to the nucleus to phosphorylate various transcription factors regulating specific gene expression involved in cell growth, differentiation, cytokine production, survival, and apoptosis. The figure was created with permission in Biorender (BioRender.com) based on the KEGG database and previous publications [73,74,75,76,77,78,79,80,81,82,83,84,85,86,87,88,89,90].

In mammalian cells, MAPK pathways are characterized and classified based on the isoforms with a similar activation motif, structure, and function. These include the classical MAPK, or Extracellular Signal-Regulated Kinase (ERK1/2), C-Jun N-terminal Kinase 1, 2, 3/Stress-Activated Protein Kinase (JNK/SAPK), and p38 kinase (p38α/β/γ/δ) [58,59,69,70,71]. Despite the unique transcriptional profile and tissue-specific expression of each isoform in various organs, pathway activation solely depends on extracellular stimuli such as growth factors, hormones, pro-inflammatory stimuli, and cellular as well as environmental stresses [59]. The activation of the MAPK-wiring network requires sequential dual phosphorylation and results in the activation of one of the 17 MAPKKKs (MAP Kinase Kinase Kinase), 7 MAPKKs (MAP Kinase Kinase), and 12 MAPKs [70,72]. MAPK protein phosphatases (MKPs) can inactivate the MAPK pathway by dephosphorylating threonine and tyrosine residues on MAPKs [59] (Figure 2).

### 3.1. ERK1/2 Pathway

The ERK1/2 pathway plays a crucial role in cell proliferation, growth, and differentiation [91,92]. This signaling transduction cascade is initiated when extracellular stimuli in the form of hormones, pro-inflammatory cytokines, and growth factors activate the transmembrane glycoproteins of designated cellular surface receptors [58,69]. These receptors include G-protein-coupled receptors (GPCRs), receptor tyrosine kinases (RTKs), and epidermal growth factor receptors (EGFRs) [58,69]. The transcriptional and translational regulation of effector genes is accomplished by downstream signal transduction through the autophosphorylation of cytosolic intermediates [58,59]. The ERK1/2 pathway also leads to the activation of the PI3K-AKT-mTOR cascade, which influences metabolic signaling and protein synthesis to sustain cell growth [58].

Upon activation, the conformational change in EGFR results in the binding of a second tyrosine kinase receptor (HER2, also known as erbB-2) to EGFR, which transphosphorylates the EGFR intracellular domains [93,94,95,96]. The activated EGFR and HER2 subsequently recruit the EGFR-associated nucleotide exchange factor Son of Sevenless (SOS) and GRB2, which act as docking sites for RAS and mediator proteins for signal transmission from receptors to soluble intracellular proteins, respectively [93,94,95,96]. The RAS superfamily of GTPases is comprised of over 150 small G-proteins, such as HRAS, KRAS, and NRAS, and is the first line of cytosolic intermediates that activate the phosphorylation cascade of the MAPK network [58]. Upon RAS binding, GDP present in the cofactor-binding site is exchanged with GTP to activate RAS [58]. Activated RAS dissociates from the activation complex to either activate various cytoplasmic proteins or stay attached to the cell membrane [93]. Hence, multiple RAS proteins can be activated by a single GRB2-SOS complex to amplify the signal [93].

The activated RAS complex recruits and interacts with the serine/threonine-protein kinase Raf (Raf-1, A-Raf, and B-Raf), leading to pathway progression by activating MEK and ERK1/2 through phosphorylation [58,69]. Cell apoptosis can also be triggered by the binding of Raf-1 to mammalian sterile 20-like kinase 2 (MST2) and apoptosis signal-regulating kinase (ASK1) [69]. Activated MEK and ERK1/2 play a crucial role in the regulation of gene expression, depending on their phosphorylated targets and irrespective of the cellular location [58]. In the cytoplasm, ERK1/2 regulates cell-functioning factors by activating rpS6, elF4B, Filamin A, and IKB-α and inhibiting eEF2K, GSK3, DAPK, METTL1, BAD, and nNos [58,97]. While in the nucleus, it regulates transcription factors by activating a wide range of factors such as Elk-1, Fos, Myc/N-Myc, CREB, ATF1, Histone H3, SRF, and many more, and inhibiting FoxO3, MKP1/2, PPARγ, and p27 KIP1 [58,97].

In the injured spinal cord, the ERK pathway leads to astrocyte proliferation and scar formation at the lesion site, which inhibits neuronal growth and axon regeneration [61,98]. Hindering the MAPK signaling pathway (using TLR9 antagonists, MEK/ERK inhibitors U0126, Ca^2+^ channel blockers, or EGFR blockers C225 and AG1478) inhibits the proliferation and migration of astrocytes, which attenuates apoptotic death in proximal axons but does not promote axonal re-growth in the astrocytic scar [61,98,99,100]. Despite the lack of axonal regeneration, the corresponding reduction in inflammatory cell infiltration, cytokine production, and microglial activation achieved by inhibition of the MEK/ERK signaling pathway leads to improved functional recovery [61].

### 3.2. SAPK/JNK Pathway

Stress-activated protein kinases (SAPKs)/Jun amino-terminal kinases (JNKs) are activated following a variety of cellular stresses and extracellular stimuli [101]. These kinases are present ubiquitously in the human spinal cord. The role of JNK in spinal cord regulation was previously reviewed by Schellino et al. 2019 [102]. SAPK-α/JNK2, SAPK-β/JNK3, and SAPK-γ/JNK1 encode for SAPKs/JNKs, which are activated through pro-inflammatory stimuli like cytokines as well as cellular and environmental stresses [103,104]. This leads to downstream signal transduction from the growth factor, major histocompatibility complex (MHC), and cytokine receptors to MAP4Ks/MAP3Ks by dual autophosphorylation [71,105].

The MAP4K proteins are upstream activators of MAP3K levels that include: Hematopoietic Progenitor Kinase-1 (HPK1), GCK-Like Kinase (GLK), Germinal Center Kinase (GCK), HPK/GCK-like Kinase (HGK), GCK-Related Kinase (GCKR), and kinase homologous to Ste20/Sps1 [71,105]. HPK1, a 97-kDa serine/threonine kinase, is associated with oncogenes Crk and CrkL, as well as GRB2 to regulate activation of TAK1 and MEKK1 in the kinase signaling cascade [71,105]. HPK1 is activated by the EGF receptor, whereas specific JNK activators, such as GLK, GCK, HGK, and GCKR, are activated by tumor necrosis factor receptors (TNFRs) [71,105].

MAP3Ks, including MAPK/ERK Kinase Kinases (MEKKs), Tumor Progression Locus-2 (TPL2), Mixed Lineage Kinase-2/3 (MLK2/3), TGF-Beta-Activated Kinase-1 (TAK1), Apoptosis Signal-regulating Kinase-1 (ASK1), and Zipper Protein Kinase (ZPK), are capable of phosphorylating and activating downstream signal transduction proteins such as MAP2K [106]. Moreover, some of the kinases involved in MAP3K levels, such as MAP3K6, LZK, and MLK1, are known to regulate MAP2Ks, however, their own activation cascade and function remain unexplored.

The MAPK kinase (MAP2K) level comprises dual-specificity kinases MKK4/7 that phosphorylate serine and threonine residues to activate JNK1/2/3 [71,105,107]. JNKs, which are active dimers, translocate across the nuclear membrane to phosphorylate c-Jun, Activating Transcription factor 2 (ATF2), tumor suppressor p53, Nuclear Factor of Activator T-Cells (NFAT4), and MAP-kinase Activating Death Domain (MADD) to increase the gene expression of Activator Protein 1 (AP1) [71,105]. Generally, JNK/SAPK signaling promotes apoptosis as well as cell survival under certain conditions; hence, it is involved in tumorigenesis, inflammation, and development [71,105].

### 3.3. p38 Pathway

The p38 pathway is a stress-activated molecular response homologous to the JNK/SAPK signaling pathway, which is triggered by pro-inflammatory cytokines (IL-1 and TNF-α) as well as other cellular and environmental stressors [69,108,109]. Depending on the type of signaling molecule, its specific MHC and cytokine receptor sequentially activate TRADD and TRAF2 to initiate downstream signal transduction [108,109].

TAK1 is a protein kinase that plays a crucial role in transducing signals from the TGF-β receptor and phosphorylating JNK, as well as the p38 kinase pathway [108,109]. Inflammatory cytokines can stimulate their specific cytokine receptors to inhibit MAP4Ks such as GCK, GCKR, GLK, as well as HGK, and eventually inhibit TAK1 [108,109]. Activated ASK1 or MEKK1 in place of TAK1 can also crosstalk by phosphorylating MKK4/7 in the JNK pathway [69]. At the activated MAP2K level, MKK6 can phosphorylate p38-α/β/γ/δ, while MKK3 can phosphorylate p38-α/γ/δ, and MKK4 can only phosphorylate p38-α [108,109].

Activation of four well-known isoforms of p38—α/β/γ/δ—results in phosphorylation and activation of downstream kinases, such as MAPK-Activated Protein Kinase-2 (MAPKAPK2), MAPK-Activated Protein Kinase-3 (MAPKAPK3), and p38-regulated/activated protein kinase (PRAK), which alters the cytoskeleton by activating Heat Shock Proteins-25/27 (HSP25/27) antigen [108,110]. There are, however, heterogeneous activation affinities, where activation of MAPKAPK2 and HSP25/27 is accomplished primarily by p38-α/β, while p38-γ/δ causes notable upregulation of AFT2 [108,109]. p38-α can phosphorylate and activate Mitogen and Stress-induced Kinase (MSK1/2), which can also be activated by the ERK1/2 signaling pathway [111]. The p38 family affects several transcription factors through MSK1/2 activation: cAMP Response Element-Binding Protein (CREB), Signal Transducers and Activators of Transcription-1 (STAT1), Elk-1, and Max/Myc complexes [108,109,111]. Hence, the p38 signaling cascade is crucial in chromatin remodelling, transcription, and cell motility [112,113].

Targeting p38 MAPK using both inhibitors and genetic disruptions of the p38 gene has demonstrated the critical role of these proteins in the pro-inflammatory response following SCI. p38 is an upstream regulator of several inflammatory pathways that require its phosphorylation in order to activate downstream targets. Intrathecal or systematic delivery of p38 inhibitors has been shown to reduce pro-inflammatory cytokine release and several pain mediators, such as prostaglandins, in the spinal cord, resulting in reduced neuropathic pain [60].

A selective inhibitor of p38, SB203580, was shown to reduce neural apoptosis and myelin degeneration, resulting in improved hindlimb function following SCI [114]. In another study, the dose-dependent administration of SB203580 post-CCI (chronic constriction injury) of the sciatic nerve reversed CCI-induced neuropathic pain, likely through the inhibition of p38, thereby modulating CREB-dependent gene expression of inflammatory cytokines [115]. Contrary to these results, a more recent study demonstrated no significant improvement in tissue sparing or functional recovery after administration of three different doses of SB203580 post-SCI [116]. Interestingly, these studies were consistent in terms of the model of injury as well as the dosage, route, and timing of SB203580 administration. However, the inconsistent results may be due to differences in the severity of the injury, resulting in reduced efficacy of the drug [116]. It is important to note that aside from its role in inflammation, p38 MAPK is involved in pathways for growth cone formation, axon development [117], and cell differentiation [118]. Thus, inhibition of p38 may interfere with spontaneous recovery. Furthermore, downstream targets of p38, such as mitogen and stress-activated kinases (MSKs) 1 and 2, are involved in the production of IL-10, an anti-inflammatory cytokine, and of DUSP1, which deactivates p38, acting as a negative feedback loop [119]. Thus, further investigation of specific p38 inhibitors is required for the use of p38 as an effective therapeutic target.

Additionally, p38 MAPK plays a critical role in the induction of long-term depression (LTD) and long-term potentiation (LTP). LTD involves either ionotropic receptors induced through the activation of NMDARs or group I metabotropic glutamate receptors. Previous studies have shown that MAPK genes are highly expressed in the CNS [120]. The MAPK-activated protein kinase (MKs) subfamily is a downstream target of p38-MAPK, which is involved in the regulation of actin remodeling. Actin filaments are important for the maintenance and growth of dendritic spines, which are responsible for the formation of synapses.

Many emerging studies have demonstrated an association between p38 MAPK activation and the activation of various nociceptive pathways in different animal models of pain. This pain pathway is often activated by inflammatory cytokines such as IL-6, Il-1B, and TNF-a, as well as cellular stress, which activate MAPKKKs and then activate p38 MAPK via phosphorylation, allowing it to translocate to the nucleus. Nuclear p-p38 can then regulate the transcription of various genes involved in the mediation of pain [121]. Furthermore, numerous studies have demonstrated that central, systemic, or local treatment of p38 MAPK inhibitors attenuates neuropathic pain in different animal models, including those of SCI [48,122,123,124].

Interestingly, minocycline is a second-generation tetracycline with a neuroprotective effect following traumatic SCI due to its anti-inflammatory, anti-apoptotic, and antioxidant properties, some of which are primarily mediated through p38 MAPK inhibition [124,125]. Several studies have reported the efficacy of minocycline in the recovery of animal models of SCI through various mechanisms, including reduced neuronal and oligodendroglial apoptosis, inhibition of microglial activation, reduced excitotoxicity, and neutralization of free radicals. In particular, minocycline inhibits p38 phosphorylation, leading to reduced pro-inflammatory cytokine and chemokine release [124,126,127], increased production of endogenous BNDF [124], and reduced iNOS expression in reactive microglia and macrophages [128]. To date, a phase II double-blind, randomized, placebo-controlled pilot clinical trial has shown possible benefits of minocycline in subsets of SCI patients [129]. Patients treated with minocycline demonstrated significant improvements in motor recovery compared to placebo controls (*n* = 44), however, this improvement was only observed in patients with cervical, and not thoracic, SCI [129]. A phase III clinical trial was subsequently initiated (clinicaltrials.gov registration number NCT01828203), although the current status of this trial is unclear.

## 4. PI3K-AKT-mTOR Network

**Figure 3:**
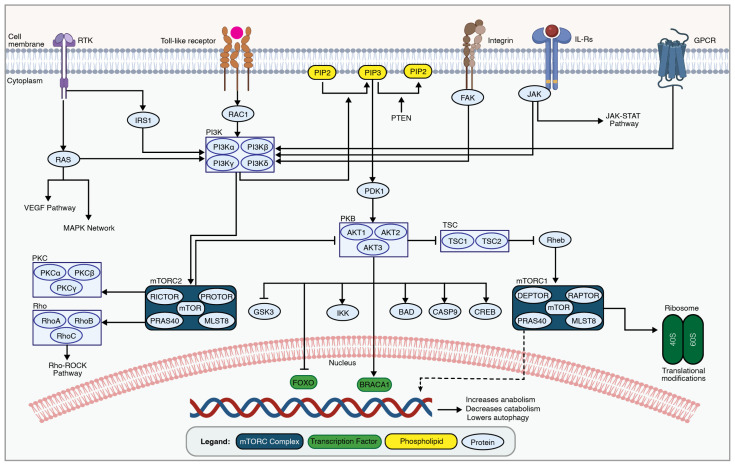
An overview of the PI3K-AKT-mTOR network. The PI3K-AKT-mTOR signaling cascade is involved in secondary SCI pathogenesis by regulating cellular metabolism, growth, and survival through a series of phosphorylation events. Transmembrane receptors, such as receptor tyrosine kinase (RTK), toll-like receptors, integrins, interleukin receptors, and GPCRs, lead to PI3K activation. This in turn phosphorylates membrane phospholipids, leading to AKT activation by phosphorylation at specific residues. Active AKT phosphorylates downstream targets, including mTOR, controlling essential cellular processes like protein synthesis and metabolism. The figure was created with permission in Biorender (BioRender.com) based on the KEGG database (Pathway IDs: hsa04151 and hsa04150) and previous publications [133,137].

The PI3K-AKT-mTOR pathway is activated by growth factors, inflammatory markers, and hormones and is involved in several cellular functions, including cell cycle regulation, cell proliferation, cellular metabolism, and apoptosis [130] (Figure 3). The PI3K-AKT-mTOR signaling network is highly conserved throughout eukaryotic evolution, and the molecular mechanism behind its function and biological role has undergone continual refinement. In addition to its involvement in the pathogenesis of secondary SCI (reviewed previously by He et al. 2022 [131] and Xiao et al. 2022 [132]), the aberrant PI3K-AKT-mTOR pathway is implicated in a variety of conditions, including cancer, chronic obstructive pulmonary disease, pulmonary fibrosis, cardiovascular disorders, and other neurological conditions [133,134,135,136].

PI3K-AKT-mTOR signaling is initiated upon extracellular signaling molecules binding to extracellular receptors, such as vascular endothelial growth factor receptor (VEGFR), B-cell receptor (BCR), interleukin-2 receptor (IL2R), and G protein-coupled receptors (GPCRs), which lead to PI3K activation [138,139,140]. Phosphoinositide 3-kinase (PI3K) is a lipid kinase that phosphorylates membrane-bound phosphatidylinositol 4,5-bisphosphate (PIP2) to generate phosphatidylinositol 3,4,5-trisphosphate (PIP3). PIP3 serves as a second messenger that recruits AKT to the cell membrane, where it undergoes activation through phosphorylation at two critical sites, Thr308 and Ser473. AKT, also referred to as protein kinase B, is a proto-oncogene as well as an ortholog of the viral v-akt and is related to protein kinase A and C serine-threonine kinases. The 3′-phosphoinositides recruit AKT to the plasma membrane via its interaction with the N-terminal region of the AKT. AKT exists in three isoforms (AKT1, AKT2, and AKT3), each with distinct functions [141]. Activated AKT phosphorylates the mammalian target of rapamycin (mTOR), which functions as a serine-threonine protein kinase incorporated either in the mammalian target of rapamycin complex 1 (mTORC1) or mammalian target of rapamycin complex 2 (mTORC2) [137]. These complexes are master regulators of cellular processes and initiate two separate signaling cascades.

The mTORC1 pathway increases cell growth, proliferation, and survival by enhancing protein, lipid, and nucleotide production and reducing autophagy. In addition to mTOR, the mTORC1 complex is composed of Regulatory-Associated Protein of mTOR (RAPTOR), G protein beta subunit-like (GβL)/mammalian lethal with SEC13 protein 8 (MLST8), DEP domain-containing mTOR-interacting protein (DEPTOR), and PRAS40 (proline-rich AKT substrate 40 kDa) [137]. In contrast, the mTORC2 pathway increases cell proliferation and survival, but its full biological function is still unknown. mTORC2 phosphorylates AKT. The constituents of the mTORC2 pathway include mTOR, GβL/mLST8, Rapamycin-insensitive companion of mTOR (RICTOR), DEPTOR, and Proline-rich protein 5 (PRR5)/protein observed with RICTOR (PROTOR) [137].

AKT also inhibits Forkhead box O (FoxO) transcription factors as well as pro-apoptotic proteins BCL2-associated agonist of cell death (BAD) and caspase 9 [142,143]. The AKT phosphorylation of FoxO transcription factors prevents their translocation to the nucleus and starts pro-apoptotic transcriptions. BAD is a proapoptotic member of the BCL-2 gene family. Caspase 9 is an initiator caspase.

Rapamycin (also known as Sirolimus) is a well-studied and multipurpose medication initially identified in fungi that targets mTOR [144]. Rapamycin is currently in the clinic as a macrolide antibiotic, cancer treatment, immunosuppressive for transplantation, and cardiovascular treatment [145]. Preclinical studies demonstrate the neuroprotective effects of rapamycin following SCI [146,147,148]. These studies demonstrate that rapamycin reduces neuronal loss in the injured spinal cord [149].

Phosphatase and tensin homolog (PTEN) is a natural inhibitor of the PI3K-AKT-mTOR pathway. PTEN is a known tumor suppressor and acts both as a lipid and protein phosphatase [150]. PTEN dephosphorylates phosphatidylinositol (3,4,5)-trisphosphate. PTEN deletion enhances mTOR activity and promotes axonal regeneration in the injured adult spinal cord via the sprouting of the uninjured corticospinal axons, which possess the ability to form synapses [151]. Similar findings have been observed in other injury models, such as optic nerve injury [152]. Recent findings demonstrate that PTEN antagonistic peptide (PAP), which blocks the PTEN’s functional domain, yields similar results and enhances axonal growth [153]. Similarly, insulin-like growth factor 1 (IGF-1) is a suppressor of the PI3K-AKT-mTOR signaling cascade and holds great promise to enhance regeneration [154,155].

## 5. Rho-ROCK Pathway

**Figure 4:**
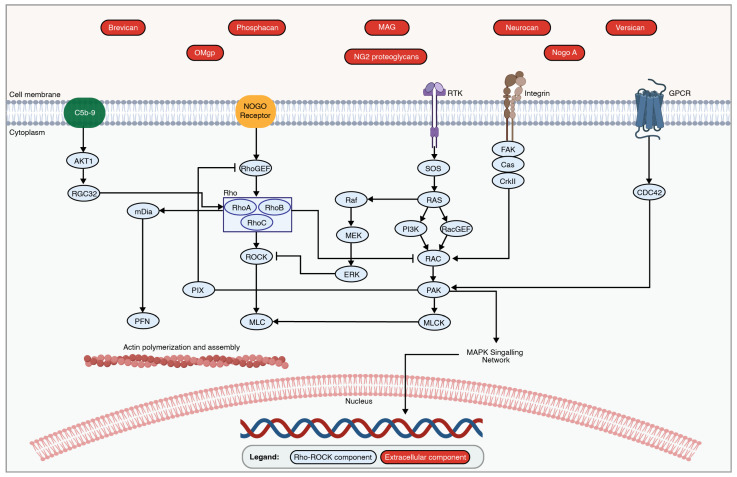
An overview of the Rho-ROCK Pathway. The binding of extracellular signals to the appropriate receptors initiates the Rho-ROCK pathway via the activation of Rho GTPases. This then leads to the activation of ROCK and the subsequent phosphorylation of downstream effectors, such as MLC. The activated Rho-ROCK pathway plays an important role in regulating actin cytoskeleton organization and cell contractility, which leads to the collapse of axonal regeneration following traumatic SCI. The figure was created with permission in Biorender (BioRender.com) based on the KEGG database (Pathway ID: hsa04810) and previous publications [159,162,174].

The Rho-ROCK pathway is an important regulator of cytoskeleton dynamics and actomyosin contractility, which plays a crucial role in controlling cellular shape, adhesion, and motility [156] (Figure 4). Following traumatic SCI, Rho-ROCK activation is a barrier to recovery, as it contributes to the collapse of axonal growth cones, failure of axonal regeneration, and neuronal loss [157]. This inhibition of axonal regeneration and its consequent attenuation of functional recovery via the activation of the Rho-ROCK pathway is instigated by many secondary extracellular signals produced in the injured spinal cord, as described below [158].

The traumatic degeneration of myelin and oligodendrocytes releases inhibitory molecules, such as neurite outgrowth inhibitor A (Nogo-A), oligodendrocyte-myelin glycoprotein (OMgp), and myelin-associated glycoprotein (MAG), which bind to Nogo receptors 1, 2, and 3 [159]. In parallel, reactive glia secrete inflammatory and inhibitory CSPGs, including brevican, phosphacan, neurocan, versican, and NG2 proteoglycans [160]. Through binding to their respective receptors, these inhibitory extracellular signals lead to the activation of Rho GTPases, which include RhoA, RhoB, and RhoC [161]. These small GTPases act as molecular switches, transitioning between an active GTP-bound state and an inactive GDP-bound state. Guanine Nucleotide Exchange Factors (GEFs) facilitate the exchange of GDP for GTP, turning on the GTPase [161]. The activated Rho GTPases, particularly RhoA, stimulate the activity of Rho-associated protein kinase (ROCK), a downstream effector in the pathway. ROCK phosphorylates various downstream targets, including myosin light chain (MLC) and myosin phosphatase, leading to the regulation of actin–myosin cytoskeletal dynamics [162]. The Rho-ROCK pathway can also influence gene expression by modulating transcription factors such as serum response factor (SRF) and myocardin-related transcription factor (MRTF) [163,164].

Cethrin (also referred to as VX-210 or BA-210) is a RhoA inhibitor that has been shown to be an effective regenerative agent in both preclinical and clinical studies of traumatic SCI. Animal studies investigating the role of Cethrin demonstrate its neuroregenerative properties and its potential to enhance functional recovery [165]. A dosage-ranging multicenter phase 1/2a clinical trial concludes that, topically, Cethrin administration on the dura mixed with fibrin sealant during decompression surgery is safe and tolerable for traumatic SCI patients [166]. Its strong efficacy for complete cervical SCI patients enticed a follow-up randomized, double-blind, placebo-controlled phase 2/3 trial, but the study was prematurely terminated at the interim efficacy-based futility analysis [167,168].

Similarly, Elezanumab is a monoclonal antibody against repulsive guidance molecule A (RGMa) that lowers Rho-ROCK signaling. Elezanumab is currently in clinical trials for multiple sclerosis (MS; clinicaltrials.gov registration numbers NCT03737851 and NCT03737812), traumatic cervical SCI (clinicaltrials.gov registration number NCT04295538), and acute ischemic stroke (clinicaltrials.gov registration number NCT04309474). RGMa is a neurite growth inhibitor that is present in either soluble or membrane-bound forms. Through interaction with neogenin and bone morphogenic protein (BMP), RGMa blocks neuroregeneration and triggers neuronal apoptosis [169]. RGMa upregulation following traumatic SCI presents a significant challenge to the regeneration of damaged neural tissue. Several animal models have been utilized to investigate the effects of RGMa inhibition on traumatic SCI recovery using either intrathecal or systemic elezanumab administration [170,171,172]. These studies demonstrate enhanced regeneration, plasticity, and repair. In primates, elezanumab echoes these findings and shows enhanced neurobehavioral recovery [169,173].

## 6. NF-κB Pathway

**Figure 5:**
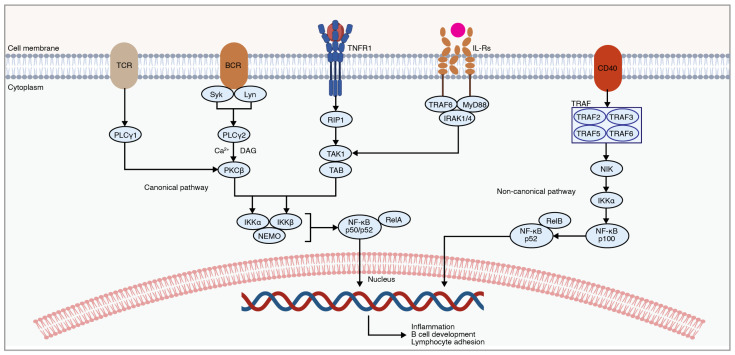
An overview of the NF-κB signaling involved in mediating the inflammatory response post-SCI. NF-κB signaling consists of canonical and non-canonical pathways. Extracellular signaling leads to the subsequent degradation of IκB proteins, resulting in NF-κB translocation into the nucleus. The figure was created with permission in Biorender (BioRender.com) based on the KEGG database (Pathway ID: hsa04064) and previous publications [176,177,189,190].

The nuclear factor kappa-light-chain-enhancer of activated B cells (NF-κB) pathway is highly intertwined due to crosstalk with the MAPK, PI3K, and JAK-STAT cascades [175]. The NF-κB pathway plays an important role in mediating the immune response, cell proliferation, and cytokine production [176]. The dysregulation of this cascade is implicated in various inflammatory and autoimmune conditions. Previous research demonstrates its involvement in the secondary neuroinflammatory response following traumatic SCI. NF-κB involves both classical and alternative pathways (Figure 5) [177]. The classical pathway (also referred to as the canonical pathway) produces a rapid and transient response by pro-inflammatory cytokines, PAMPs, and DAMPs [177]. The alternative pathway (also referred to as the non-canonical pathway) produces a slower response and is activated by a small subset of cytokines [177].

Under normal physiological conditions, the NF-κB pathway is inactive [178]. Upon binding of pro-inflammatory cytokines to extracellular receptors, including TNF receptors or interleukin receptors, downstream molecules such as protein kinase C (PKC), TAK1, TAB, and NIK are activated. These molecules lead to the activation of the inhibitory-κB kinase (IKK) complex, consisting of IKKα, IKKβ, and NEMO (also known as IKKγ). This IKK complex then phosphorylates IκBs, marking them for ubiquitination and subsequent proteasomal degradation [177]. As a result, the NF-κB complex consisting of p50 and p65 (also known as RelA) is then translocated to the nucleus [179]. In the nucleus, the NF-κB complex binds to specific DNA sequences, known as κB sites, which regulate the transcription of specific target genes involved in immune and inflammatory responses, cell survival, proliferation, and differentiation [179]. These genes include pro-inflammatory cytokines and chemokines, adhesion molecules, and anti-apoptotic proteins [180].

Histological analysis demonstrates NF-κB activation in the injured spinal cord [181]. Western blotting and immunohistochemical staining illustrate the presence of activated p65 in the NF-κB dimer as early as 0.5 h post-SCI and persist until 72 h after injury [181]. Cellular staining demonstrates that activated NF-κB signaling is present in microglia, endothelial cells, and neurons [181]. Interestingly, several natural compounds have been shown to protect the injured spinal cord from SCI-induced inflammatory responses through NF-κB signaling attenuation [175]. These include Resveratrol [182], Forsythiaside B [183], Geniposide [184], Wogonoside [185], Sesamol [186], Curcumin [187], and Triptolide [188].

## 7. JAK-STAT Pathway

**Figure 6:**
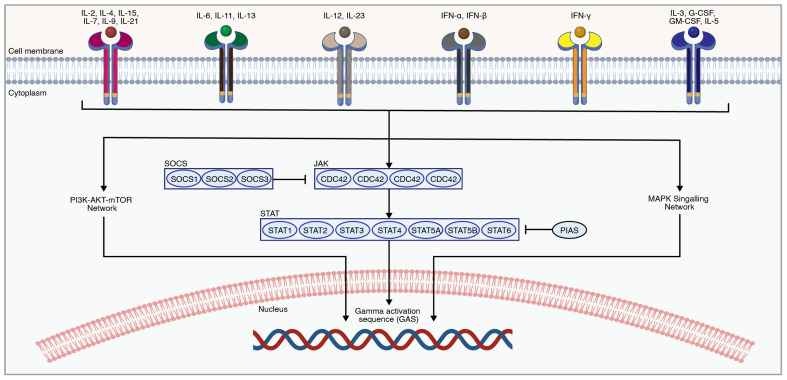
An overview of the JAK-STAT pathway. Extracellular ligands activate-related receptors to transphosphorylate and then phosphorylate downstream molecules such as STATs, which ultimately cause changes in the transcriptome [202]. The figure was created with permission in Biorender (BioRender.com) based on the KEGG database (Pathway ID: hsa04630) and previous publications [191,203].

The Janus kinase/signal transducer and activator of transcription (JAK/STAT) is involved in cell-cycle functions such as cellular division, proliferation, and apoptosis, as well as various biological processes such as hematopoiesis, scar formation, and immunological response (Figure 6). The role of the JAK/STAT pathway in SCI was previously reviewed by Guo et al. 2023 [191].

JAK-STAT is activated through the binding of extracellular cytokines or growth factors to their cell surface receptors, which leads to the activation of Janus kinases (JAKs) through autophosphorylation. Phosphorylated JAKs in turn phosphorylate signal transducer and activator of transcription (STAT) proteins, which will then dimerize and translocate into the nucleus to induce a transcriptional response. The JAK family of non-receptor tyrosine kinases consists of four proteins, including JAK1, JAK2, JAK3, and TYK2 [192]. JAKs activate their downstream molecules, known as STAT proteins. The STAT family members include STAT1, STAT2, STAT3, STAT4, STAT5A, STAT5B, and STAT6 [192]. STATs can also be activated by SRC family kinases [193]. Phosphorylated STATs undergo conformational changes, allowing them to form homo- or hetero-dimers through reciprocal interactions between their Src homology 2 (SH2) domains, which are then translocated to the nucleus to alter the transcriptional pattern.

The JAK-STAT pathway plays several important roles in the pathogenesis of secondary SCI. First, it mediates the astrocytic response after SCI, including the formation of the astrocytic barrier and protein secretion. Second, the JAK-STAT pathway alters inflammation [194]. Third, it alters the neuronal response. A recent study demonstrated that acute production of IL-6 in a mouse SCI model leads to the activation of JAK-STAT signaling in neurons, particularly through JAK1 phosphorylation at the Tyr1022/1023 residue [195]. Interestingly, the JAK inhibitor AG-490 suppressed JAK1 phosphorylation and reduced functional recovery [195]. Fourth, it mediates the differentiation of neural progenitor cells toward an astrocytic lineage [196].

Suppressor of Cytokine Signaling (SOCS) proteins are major regulators of the JAK-STAT pathway. SOCS3 is a negative regulator of the JAK/STAT pathway and plays an important role in modulating inflammation and the cellular response to cytokines [197,198]. SOCS3 deletion in SCI results in increased axonal sprouting in the spared corticospinal tract and leads to improved recovery in a unilateral pyramidotomy SCI model [199].

A recent transcriptional analysis in *Xenopus laevis* tadpoles highlights the importance of the JAK-STAT pathway for spinal cord regeneration and demonstrates its differential regulation between regenerative and non-regenerative stages [200]. Following SCI, regenerative tadpoles quickly activate this pathway transiently, while non-regenerative stages show delayed and sustained activation. Additionally, STAT3, a key player in this pathway, becomes active mainly in Sox2/3+ ependymal cells, motoneurons, and sensory neurons post-injury. Manipulating STAT3 activation reveals its significant role in controlling the expression of pro-neurogenic genes after injury. This highlights the crucial involvement of the JAK-STAT pathway in regulating neural progenitor fate during spinal cord regeneration in tadpoles [200,201].

## 8. Translational Implications and Future Directions

Due to the complexity and multifaceted nature of secondary traumatic SCI pathogenesis, timely intervention is crucial to mitigate further secondary damage and enable enhanced recovery [13,204,205,206]. Recent advances in molecular technologies have provided major milestones in developing therapeutics that can alter the secondary pathogenesis and target the molecular cascades within the MAPK, PI3K-AKT-mTOR, JAK-STAT, and Rho-ROCK signaling networks. Targeting these cascades can alter neuroinflammation, apoptosis/cell death, cellular proliferation, tissue repair, and neuronal regulation. While many pharmacological agents that target these pathways have been investigated for traumatic SCI, important factors such as drug penetrance across BSCB, potency and effectiveness, toxicity or adverse off-target effects, and drug metabolism limit their efficacy for clinical translation.

The heterogeneity of traumatic SCI cases is another important difficulty in developing efficacious pharmacological interventions [207,208]. For instance, the pathogenesis and incidence of traumatic SCI vary depending on the injury level. Epidemiologically, SCI occurs most frequently at the cervical level, followed by the thoracic and lumbosacral levels [25]. Cervical SCI patients consequently exhibit tetraplegia—an impairment of all four limbs—which severely impacts their quality of life [209]. Additionally, damage to the cervical autonomic tracts, phrenic neurons, and sympathetic preganglionic neurons (SPNs), further complicates this condition, leading to dysfunctional breathing [210], autonomic dysreflexia [20,211], impaired cardiovascular function, and secondary immunodeficiency [212]. Many pharmacological interventions demonstrate different efficacy for improving functional recovery depending on the level of injury.

Considering the complex and interconnected network of intracellular biochemical events triggered following traumatic SCI, protein kinases are promising targets to inhibit the progression of secondary SCI damage. These globular enzymes regulate cell signaling and gene expression in the local and adjacent cells of the injured spinal cord. Notably, midostaurin is a small-molecule pan-protein kinase inhibitor that can cross the BSCB and inhibit a vast array of protein kinases through competitive binding to ATP binding sites. A recent investigation of midostaurin demonstrates its ability to ameliorate the early secondary injury responses seen following traumatic cervical SCI and improve functional recovery, which presents a viable neuroprotective approach for combating the secondary injury response present after SCI [213].

Alternatively, combined treatments for traumatic SCI offer a multifaceted approach, integrating various therapeutic interventions to enhance recovery. Recent studies have demonstrated how a combination of different treatments, such as stem cell therapy, rehabilitative training, or electrical stimulations, can work synergistically to enhance functional recovery [214,215,216]. For SCI-induced intracellular signaling cascades, while this can be achieved by the administration of multiple pharmacological drugs, advances in gene therapy enable precise targeting of multiple intracellular substrates. The clinical approval of AAV9-based therapeutics for spinal muscular atrophy in 2019 has opened many possibilities for gene therapies for the treatment of traumatic SCI [214,217]. An AAV-based combined approach in transgenic mice, targeting SOCS and PTEN, shows great effectiveness in improving functional recovery [199]. In another study, a combined AAV strategy was used to induce axonal growth by enabling intrinsic growth capacity in neurons, growth-supportive substrates, and chemoattraction through PTEN, IGF1, CNTF, laminin, FGF2, EGF, and GDNF manipulation [218]. This combined strategy demonstrates improved propriospinal axonal regrowth by 100-fold in the injured spinal cords of rats and mice [218]. Such combined treatment strategies hold great promise for clinical translation and improving recovery for patients with traumatic SCI.

Future studies establishing the cell-specific role and impact of selective modulation of the intracellular signaling cascades could uncover the complex molecular mechanisms that regulate this promising therapeutic target for traumatic SCI. Recent advances in single-cell RNA-sequencing and single-nucleus RNA-sequencing transcriptional analyses enable more precise analyses [219]. ScRNA-Seq refers to the isolation and sequencing of the total RNA extracted from each cell, which enables the investigation of gene expression patterns in each cell and cellular heterogeneity. SnRNA-Seq investigates the nuclear RNA, excluding the cytoplasmic RNA [219]. These transcriptional profiling tools enable analysis of differential expression of protein kinases in each particular cell type, kinome-based cellular diversity, characterization of cell states, and investigation of cellular interaction.

In addition to transcriptional analyses, future investigations using mass-spectrometry-based phosphoproteomics as well as high-throughput kinome-wide screens can enable closer examination of kinase activity, function, and interaction [220,221,222]. Mass-spectrometry enables the identification of the interactome of proteins in cells. Kinome-wide screens comprehensively assess the inhibitory outcome of a wide range of compounds against a diverse set of kinases. These can include the incorporation of RNAi and CRISPR. These techniques offer a promising avenue to uncover the full therapeutic potential of the intracellular signaling pathways following a traumatic spinal cord injury.

## 9. Conclusions

The MAPK, PI3K-AKT-mTOR, Rho-ROCK, NF-κB, and JAK-STAT signaling cascades consist of groups of serine–threonine kinase proteins, which are crucial for the propagation of the secondary SCI pathogenesis. These signaling cascades are involved in multiple secondary processes after traumatic SCI, including immune responses, scar formation, and neuroplasticity. While some of these mechanisms are considered an adaptive response to the initial injury, their continued activation results in further spinal cord injury. Therapeutic targeting of these signaling pathways presents a promising strategy to avoid the deleterious impact of these mechanisms. The diverse set of pharmacological candidates that target these intracellular networks shows great promise for improving recovery post-SCI. Opportunities for further drug development to examine this promising target exist and could lead to novel translational opportunities.

## Data Availability

All inquiries should be directed to the corresponding author.
